# USP7 Inhibits Osteoclastogenesis *via* Dual Effects of Attenuating TRAF6/TAK1 Axis and Stimulating STING Signaling

**DOI:** 10.14336/AD.2023.0325-1

**Published:** 2023-12-01

**Authors:** Ziang Xie, Yizheng Wu, Yang Shen, Jiandong Guo, Putao Yuan, Qingliang Ma, Shiyu Wang, Zhiwei Jie, Hongyi Zhou, Shunwu Fan, Shuai Chen

**Affiliations:** ^1^Department of Orthopedic Surgery, Sir Run Run Shaw Hospital, Zhejiang University School of Medicine, Hangzhou, China.; ^2^Key Laboratory of Musculoskeletal System Degeneration and Regeneration Translational Research of Zhejiang Province, Hangzhou, China.; ^3^Department of Orthopedic Surgery, Ninth people’s Hospital of Hangzhou, Hangzhou, China.

**Keywords:** Osteoclast, Ubiquitin specific protease 7, TRAF6, STING

## Abstract

Ubiquitination is a reversible post-translational modification implicated in cell differentiation, homeostasis, and organ development. Several deubiquitinases (DUBs) decrease protein ubiquitination through the hydrolysis of ubiquitin linkages. However, the role of DUBs in bone resorption and formation is still unclear. In this study, we identified DUB ubiquitin-specific protease 7 (USP7) as a negative regulator of osteoclast formation. USP7 combines with tumor necrosis factor receptor-associated factor 6 (TRAF6) and inhibits its ubiquitination by impairing the Lys63-linked polyubiquitin chain. Such impairment leads to the suppression of receptor activator of NF-κB ligand (RANKL)-mediated nuclear factor-κB (NF-κB) and mitogen-activated protein kinases (MAPKs) activation without affecting TRAF6 stability. USP7 also protects the stimulator of interferon genes (STING) against degradation, inducing interferon-β (IFN-β) expression in osteoclast formation, thereby inhibiting osteoclastogenesis cooperatively with the classical TRAF6 pathway. Furthermore, USP7 inhibition accelerates osteoclast differentiation and bone resorption both in vitro and in vivo. Contrarily, USP7 overexpression impairs osteoclast differentiation and bone resorption in vitro and in vivo. Additionally, in ovariectomy (OVX) mice, USP7 levels are lower than those in sham-operated mice, suggesting that USP7 plays a role in osteoporosis. Altogether, our data reveal the dual effect of USP7-mediated TRAF6 signal transduction and USP7-mediated protein degradation of STING in osteoclast formation.

## INTRODUCTION

The ubiquitin (Ub)-proteasome system is mainly composed of Ub, Ub-activating enzyme (E1), Ub-conjugating enzymes (E2s), Ub-protein ligating enzymes (E3s), 26S proteasome, and deubiquitinases (DUBs) [1, 2]. Ub is a highly conserved small polypeptide composed of 76 amino acid residues, encoded by several genes [3]. E1 activates Ub through the formation of a thioester bond between the carboxyl end of Ub and the activated cysteine. Activated Ub is then covalently bound to E2 binding enzyme, to degrade the ubiquitinated proteins in the cytoplasm or nucleus. This process requires E3 cooperation, which is responsible for ubiquitinating the substrate protein [1, 4]. DUB is an isomer of cysteine proteases that acts as a scissor, separating the bonds between Ub and the substrate protein and the Ub from the poly-Ub chains [5]. DUBs have been implicated in cell differentiation, homeostasis, and organ development [6]; however, the role of DUBs in bone homeostasis, as well as its underlying mechanisms, are still unclear, particularly in bone-resorbing osteoclasts.

Osteoclasts are multinucleated cells that degrade the bone matrix, leading to the loss of bone microstructure and mechanical properties [9]. Excessive bone resorption contributes to several bone diseases, such as rheumatoid arthritis, bone erosion, and osteoporosis [7, 8]. Macrophage colony-stimulating factor (M-CSF) and receptor activator of nuclear factor-κB (RANK) ligand (RANKL) are essential cytokines during osteo-clastogenesis [10]. Previous studies have shown that tumor necrosis factor receptor-associated factor 6 (TRAF6), a well-known E3, is critical for RANK signaling in osteoclastogenesis [11, 12]. The activity of TRAF6 is mediated through ubiquitination [13]. Generally, Ub molecules are linked through 48 or 63 lysine residues (known as Lys48 and Lys63) [14], forming Ub-polychains with different effects on the substrate proteins. A protein with Lys48 polyubiquitination (PUB) is mainly marked for degradation through the 26S proteasome, whereas a protein with Lys63 PUB usually targets downstream signal transduction, such as activation of kinases, alteration of protein intracellular locations, and DNA repair [15].

RANKL binds to RANK to mediate downstream signaling, which promotes the Lys63 PUB [16]. Previous studies have shown that DUBs reverse Lys63-linked TRAF6 PUB, like A20 and cylindromatosis (CYLD). These two proteins target TRAF6 for deubiquitination and suppress downstream signaling events for osteoclastogenesis [17, 18]. These studies highlight the critical role of DUB as a key regulatory mechanism of TRAF6 activity in osteoclasts. Moreover, a negative feedback pathway is also critical for osteoclastogenesis, in which the upregulation of IFN-β binding to its receptor induces downstream inhibition of osteoclastogenesis [19]. Type I interferon (IFN), such as IFN-β, is produced by the activation of a stimulator of interferon genes (STING) [20]. STING activates TANK-binding kinase 1 (TBK-1) that promotes phosphorylation of IFN regulatory factor 3 (IRF3), ultimately inducing IFN-β expression [21]. IFN-β exerts anti-leukemic effects through the promotion of dendritic cell maturation and T-cell activation [22]. However, the role of STING-induced IFN-β expression in osteoclasts remains poorly understood.

The ubiquitin-proteasome system (UPS) plays a critical role in bone homeostasis [23]. We previously found that p38/MAPK inhibition promotes ubiquitination-mediated degradation of β-catenin, leading to the suppression of osteoclastogenesis [24]. In this study, we aimed to further identify whether other DUBs are involved in the regulation of osteoclastogenesis and to explore whether these enzymes could be potential therapeutic targets. Here, we hypothesized that ubiquitin-specific protease 7 (USP7) plays an important role in the regulation of osteoclastogenesis via hybrid mechanisms. The results show that USP7 negatively regulates osteoclastogenesis via inhibiting signal transduction of TRAF6 and protecting STING from degradation.

## MATERIALS AND METHODS

### Reagents

P5091 was obtained from Selleck (Shanghai, China). DMSO was purchased from Sigma-Aldrich (St. Louis, MO, USA). CCK-8 (Cell Counting Kit-8) was obtained from Dojindo Molecular Technology (Kumamoto, Japan). Recombinant soluble mouse M-CSF and mouse RANKL were purchased from R&D Systems (Minneapolis, MN, USA). Specific antibodies anti-phosphor-p38, anti-total-p38, anti-phosphor-ERK, anti-total-ERK, anti-phosphor-JNK, anti-total-JNK, anti-phosphor-p65, anti-total-p65, anti-phosphor-TAK1, anti-total-TAK1, anti-IκBα, anti-TRAF6, anti-USP7, anti-Ubiquitin, anti-c-Fos, anti-NFATc1, anti-TRAP, anti-Flag, anti-HA, anti-Histone, anti-Myc, anti-β-actin and anti-β-tubulin were obtained from Cell Signal Technology (Shanghai, China) and Abcam (Shanghai, China), the detailed information of antibodies was shown in Supplementary Table 1. TRAP (tartrate-resistant acid phosphatase) staining kit and all other reagents were purchased from Sigma-Aldrich (St. Louis, MO, USA), unless otherwise stated.

### BMMs preparation and osteoclast differentiation

Primary bone marrow derived macrophages (BMMs) were isolated from the whole bone marrow of male 6-week-old C57BL/6 mice as described previously [29]. Cells were isolated from the femoral and tibial bone marrow and cultured in α-MEM supplemented with 10% FBS, 1% penicillin/streptomycin, and 25 ng/mL M-CSF in an incubator with 5% CO_2_ at 37°C for about 4 days until they reached 90% confluence. The BMMs were seeded into 96-well plates at a density of 8×10^3^ cells/well, in triplicate, in the presence of 25 ng/mL M-CSF, 50 ng/mL RANKL, and different concentrations of P5091, siRNA or shRNA, over expression plasmid or lentivirus. Untreated cells were included as controls. The culture medium was replaced every 2 days and osteoclasts were generally cultured for 5 days. Afterwards, the cells were washed twice with PBS, fixed in 4% paraformaldehyde for 15 min, and stained for TRAP. TRAP-positive cells with 5 nuclei and more than 5 nuclei were considered as osteoclasts.

### Bone resorption assay

BMMs were seeded at a density of 2×10^4^ cells/well in the presence of 25 ng/mL M-CSF and 50 ng/mL RANKL for 4 days on bone slices, in triplicate, transfected by siRNA or plasmid, and maintained for the additional 5 days. Untreated cells were used as a control. Resorption pits were imaged using a scanning electron microscope (FEI Quanta 250) and the bone resorption area was quantified using ImageJ software (NIH, USA).

### RNA extraction and quantitative real-time PCR

RNA extraction and quantitative Real-time PCR assay were performed according to the previous study [34]. Specificity of amplification was verified by performing RT-PCR and analyzing the melting curves. Mouse *Gapdh, Ctsk, Acp5, c-Fos, Nfatc1 and Usp7* primer sequences are presented in Supplementary Table 2.

### In vitro gene knockdown and overexpression experiments

When the BMMs reached 70%-80% confluence or were induced with M-CSF and RANKL on bone slices for 5 days, cells were transfected with 10 nM siRNA, 10 nM shRNA or 1 ng overexpression plasmid using Lipofectamine 3000 for 12 hours. After medium was changed, cells were incubated overnight before RANKL treatment or incubated with M-CSF and RANKL for further bone resorption. The sequences of siRNA and shRNA were shown in Supplementary Table 3.

### Western blot

According to our previous study [24], cells were incubated in RIPA buffer (Cell Signaling Technology, Boston, MA, USA) mixed with 100 mM phenylmethanesulfonyl fluoride (PMSF) and phosphatase inhibitor (Cell Signaling Technology, Boston, MA, USA) on ice, followed by centrifugation at 12,000 rpm for 15 mins to isolate the supernatant for the next step. Proteins were resolved by 10% SDS-PAGE and transferred to PVDF membranes (Bio-Rad) by electroblotting (250 mA, 120 mins). The membranes were blocked with 5% non-fat dry milk in TBST solution at room temperature for 1 h and then incubated with the indicated primary antibodies overnight at 4°C. Protein bands were developed by using a horseradish peroxidase-conjugated goat-anti-rabbit or goat-anti-mouse immunoglobulin G (Abcam), followed by detection with ECL reagent (Millipore, Billerica, MA, USA) or ECL reagent (FUDE BIOLOGICAL TECHNOLOGY, Hangzhou, China). Protein bands were visualized using LAS-4000 Science Imaging System (Fujifilm, Tokyo, Japan), and the obtained images were analyzed with the ImageJ software (National Institutes of Health, Bethesda, MD, USA).

### Co-immunoprecipitation (IP) assay

Both primary cells and 293T cells were used for co-IP assay. Cell extracts were lysed by the mixture solution with 1% NP-40 and 0.25% sodium deoxycholate. The lysis was firstly precleared with 25 μl of protein A/G-agarose (50% v/v). The supernatants were immunoprecipitated with 2 μg of anti-TRAF6, 1 μg anti-Flag or 1 μg anti-HA antibodies for overnight at 4°C, followed by incubation with protein A/G-agarose 4 h at 4°C. Protein A/G-agarose-antigen-antibody complexes were collected by centrifugation at 12,000 rpm for 60 s at 4°C. The pellets were washed five times with 1 mL of IPH buffer (50 mM Tris-HCl, pH 8.0, 150 mM NaCl, 5 mM EDTA, 0.5% Nonidet P-40, and 0.1 mM PMSF), for 5 min each time at 4°C. Bound proteins were resolved by SDS-PAGE, followed by western blotting with anti-Ubiquitin, anti-USP7, anti-K63, anti-K48, anti-HA, anti-Flag, anti-Myc, anti-GAPDH antibodies. The experiments were replicated for three times.

### Luciferase reporter genes assay

pNFκB-luc and pNFATc1-luc were provided by GenePharma, Shanghai. Briefly, RAW264.7 cells were seeded into the 24-well plates overnight. Then, the cells were stimulated with RANKL or with different concentrations of P5091. After 24 hours, the light intensity levels of cells were detected by One-Lumi™ Firefly luciferase reporter gene Kit (Beyotime, Shanghai, China).

### Immunofluorescence staining

For in vitro immunofluorescence staining assay, RAW264.7 cells were seeded at a density of 1×10^4^ cells in 96-well plates. After treated with shUSP7 for 12h or with P5091 for 24h, the cells were stimulated with RANKL for 15 mins, or the cells were treated with RANKL for 24h to test the localization of USP7 and TRAF6. After incubation, cells were washed twice with PBS, fixed in 4% paraformaldehyde for 30 minutes and then washed with PBS three times before permeabilization with 0.1% Triton-X100 for 30 minutes at room temperature. After blocking with 5% goat serum with PBS solution for 1 hour at room temperature, cells were incubated with anti-p65 antibody diluted 1:200 in 1% BSA-PBS, anti-USP7 antibody diluted 1:100 in 1% BSA-PBS, anti-TRAF6 antibody diluted 1:200 in 1% BSA-PBS at 4 °C overnight. Nuclears were stained with 0.1 μg/mL DAPI (Sigma-Aldrich) in PBS at room temperature for 10 minutes in the dark condition. After being washed three times with PBS, the cells were imaged using fluorescence microscope MODEL BX51TRF (OLYMPUS CORPORATION, Tokyo, Japan) and the quantification analysis of image was performed by Image J software (National Institutes of Health, Bethesda, MD, USA).

For in vivo immunofluorescence staining assay, The number of TRAP and USP7 positive cells normalized to the bone parameter (N. TRAP^+^ cells /B.Pm, N. USP7^+^ cells /B.Pm) were analyzed in each sample. The quantification of image was analyzed by Image J software (National Institutes of Health, Bethesda, MD, USA).

### In vivo bone loss experiments

Ten-week-old female C57BL/J6 mice were implanted with collagen sponges soaked with PBS or RANKL (1 μg) in the middle of calvariae as previously described [24]. The sh-USP7 or Lenti-USP7 (GenePharma, Shanghai, China) were injected onto calvariae twice, as indicated times. After 10 days, calvariae were subjected to micro-computed tomography (micro-CT) and histological analyses. Animal experiments were approved by the Committees on the Care and Use of Animals in Research at Sir Run Run Shaw Hospital. All the animals were kept in an SPF facility with a 12-hour light/dark cycle.

**Figure 1.**
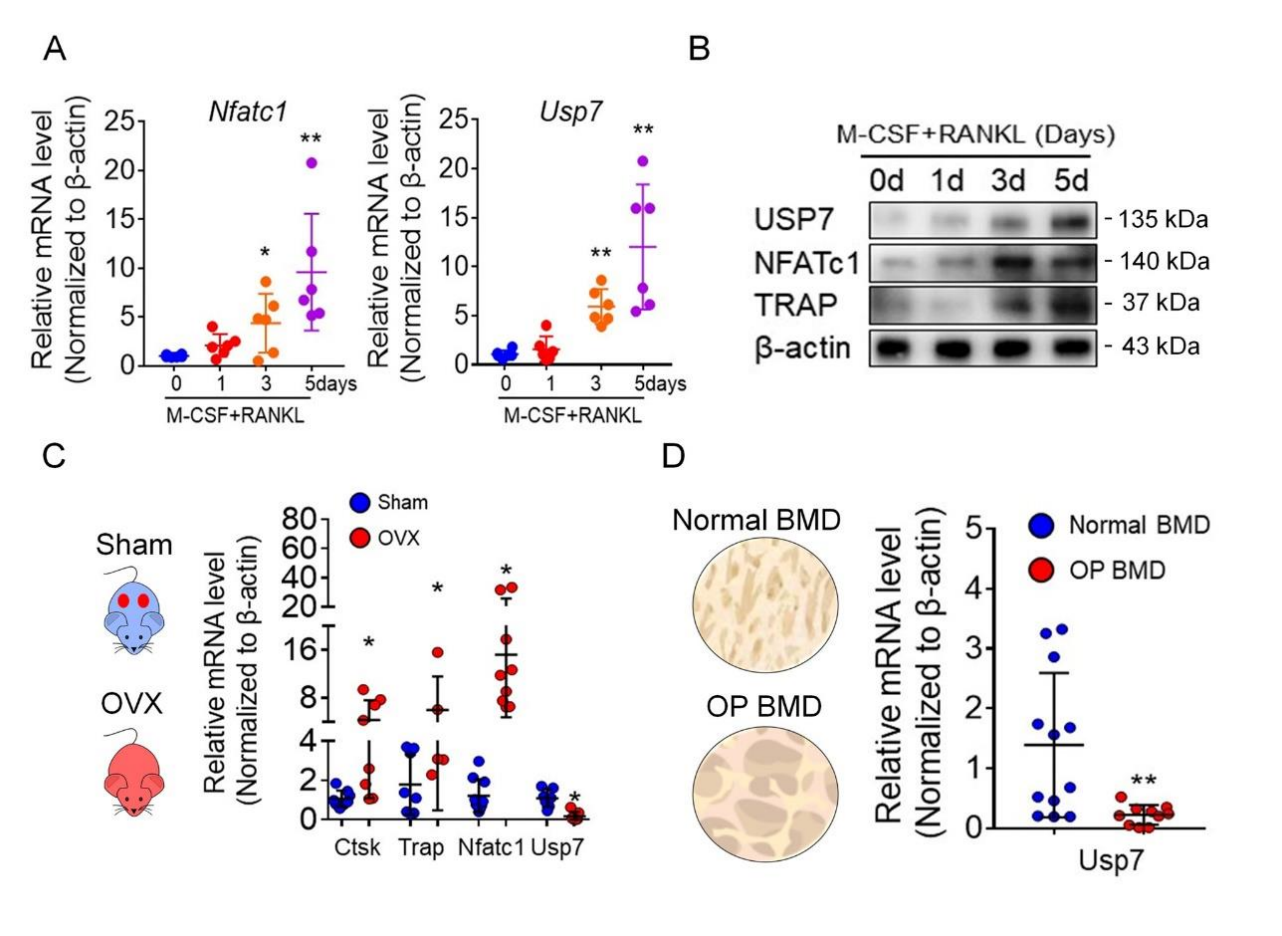
The alteration of USP7 in osteoclastogenesis and osteoporosis samples. (A) The mRNA levels of Nftac1 and Usp7 in different stages of osteoclast formation analyzed by qPCR assay. Data are presented as the mean ± SD. *, P < 0.05. **, P<0.01. n=6. Student’s *t*-test analysis was used. (B) The protein levels of USP7, NFATc1 and TRAP during osteoclast formation analyzed by Western blot assay. Cells were lysed for performing western blot assay with the indicated antibodies. Blot assays were repeated three independent experiments. (C) The mRNA levels of Nftac1, Ctsk, Trap and Usp7 in femurs of sham and OVX mice analyzed by qPCR assay. Data are presented as the mean ± SD. *, P < 0.05. n=8 in each group. Student’s *t*-test analysis was used. (D) The mRNA levels of Usp7 in cancellus bone of femoral head from normal BMD and osteoporosis patients (T<-2.5) analyzed by qPCR assay. Data are presented as the mean ± SD. *, P < 0.05. n=12 in normal BMD group, n=10 in osteoporosis BMD group. Student’s *t*-test analysis was used.

### In vivo osteoporotic mice model

12-week-old female C57BL/J6 mice were purchased from the Laboratory Animal Center of Sir Run Run Shaw Hospital (n=6 per group). Mice were maintained in a pathogen-free facility on a 12-h light/dark cycle with water and food provided adlibitum. Mice were sham-operated (Sham) or ovariectomized (OVX). The OVX mice presented osteoporosis as described previously [34]. Mice were allowed 2 months to recover from the ovariectomy surgery and then sacrificed for the related assays. To evaluate the mass and microarchitecture of the bone between different groups, micro-CT was performed. The parameters of bone volume/total volume (BV/TV) and trabecular number (Tb.N) in the trabecular region (1-2 mm distal to the proximal epiphysis) were calculated using an Inveon Research Workplace (Siemens) according to the guidelines set by the American Society for Bone and Mineral Research. In addition, sections (5-μm thickness) of femur specimens from the different groups were stained with H&E and TRAP to evaluate bone loss and osteoclast number in OVX mice.

### Cancellus bone samples from osteoporotic patients

22 cancellus bone samples of femoral head were collective from the patients undergoing total hip arthroplasty. Normal BMD and osteoporosis were identified by the BMD analysis (T≥-1.0 to control group, T<-2.5 to osteoporosis group). The human study was approved by the Medical Ethics Committees of Sir Run Run Shaw Hospital, and written informed consents were obtained from the participants before cancellus bone collection.

### Micro-CT scanning analysis

The fixed calvarial bones and tibiae were analyzed using a high-resolution micro-CT (Skyscan 1072, Aartselaar, Belgium) instrument. After reconstruction, a square ROI (region of interest), set at 0.5 mm from the calvarial bone, was selected for further qualitative and quantitative analysis. BV/TV (trabecular bone volume per total volume) was determined for each sample, as reported previously [34].

### Bone histomorphometry analysis

Histomorphometry analysis was performed as our previous protocols reported [24]. Fixed calvarial bones and femoral bones were decalcified in 10% EDTA for 2 weeks and embedded in paraffin. Histological sections were prepared for H&E staining or TRAP staining. The ratio of N.Oc/B.Pm (the number of osteoclast/Bone parameter) and Oc.S/BS (surface area of osteoclast/bone surface area) were assessed in each sample. The quantification of image was analyzed by Image J software (National Institutes of Health, Bethesda, MD, USA).

### Statistical analysis

The data was expressed as mean ± SD. Statistical analyses were performed using Prism 7 (GraphPad Software, Inc., San Diego, CA, USA). Statistical differences of two groups were assessed by Student’s *t*-test analysis, the statistical tests used assume a normal distribution, if the data are not normal, a non-parametric alternative analysis was used. Statistical differences of multiple groups were assessed by one-way ANOVA followed by Tukey’s post hoc analysis where appropriate. P-values <0.05 were considered statistically significant.

## RESULTS

### USP7 in osteoclast differentiation and osteoporotic samples

To explore the alteration of USPs in osteoclastogenesis, we focused on the alteration of Ub-specific proteasome 7 (USP7), a potential target for cancer treatment [25]. We found an upregulation of Usp7 mRNA (Fig. 1A) and protein levels (Fig. 1B) during osteoclast differentiation. Furthermore, we also observed downregulated mRNA levels of Usp7 in OVX mice (Fig. 1C). In accordance, the analyzed cancellous bone of the femoral head from patients with osteoporosis (BMD, T<-2.5) also showed decreased Usp7 expression (Fig. 1D). Altogether, these data suggest that USP7 is upregulated during osteoclast differentiation and might be a negative regulator of osteoclastogenesis.

### USP7 knockdown and inhibition promote osteoclast formation and bone resorption, however, USP7 upregulation inhibits osteoclast formation

**Figure 2.**
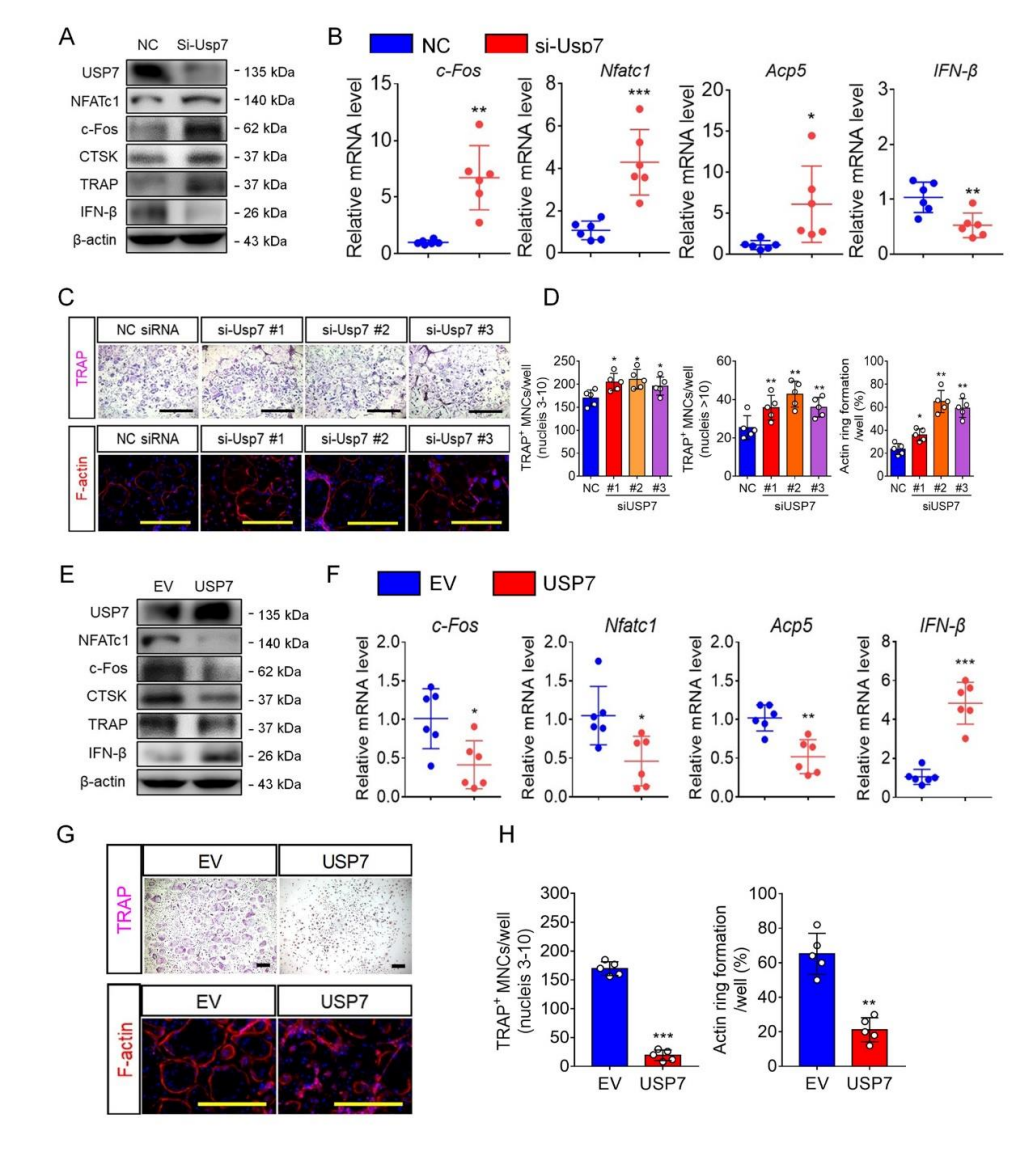
USP7 silencing promotes osteoclast formation and bone resorption. (A) Cells were harvested for performing western blot assay with antibodies against USP7, NFATc1, c-Fos, Cathepsin K, TRAP, and β-actin. Blot assays were repeated three independent experiments. (B) Quantitative RT-PCR analysis for Nfatc1, c-fos, Acp5 and IFN-β in NC siRNA-treated or Usp7 siRNA-treated cells. Data are presented as the mean ± SD. *, P < 0.05. **, P < 0.01. ***, P < 0.005. n=6 per group. Student’s *t*-test analysis was used. (C) BMMs transfected with NC siRNA or Usp7 siRNA were cultured with M-CSF and RANKL for 5 days, then cells were fixed and stained for TRAP or phalloidin. Scale bar = 100 μm. n=5 per group. (D) The number of TRAP-positive MNCs (3~10 nuclei per well or >10 nuclei per well) per well and the F-actin ring areas were analyzed. Data are presented as the mean ± SD. *, P < 0.05. **, P < 0.01. n=5 per group. Student’s *t*-test analysis was used. (E) BMMs were transfer with empty vector and Usp7 overexpression plasmid. Cells were harvested for performing western blot assay using antibodies against USP7, NFATc1, c-Fos, Cathepsin K, TRAP, and β-actin. (F) Quantitative RT-PCR analysis for Nfatc1, c-fos, Acp5 and IFN-β in EV control-transfected or USP7 overexpression vector-transfected cells. Data are presented as the mean ± SD. *, P < 0.05. n=6 per group. Student’s *t*-test analysis was used. (G) BMMs transfected with empty vector (EV) or USP7-plasmid were cultured with M-CSF and RANKL for 5 days, then cells were fixed and stained for TRAP. Scale bar = 100 μm. (H) The number of TRAP-positive MNCs (3~10 nuclei per well) per well and the area of F-actin rings were analyzed. Scale bar = 100 μm. Data are presented as the mean ± SD. **, P < 0.01. ***, P < 0.05. n=5 per group. Student’s *t*-test analysis was used.

To further explore the role of USP7 in osteoclast differentiation, we transfected BMMs with Usp7 siRNA. As shown in Figure 2A and 2B, we successfully achieved Usp7 silencing, confirmed through mRNA and protein levels, respectively. Meanwhile, the expression of OC-related genes and proteins was upregulated upon USP7 knockdown, whereas the expression of IFN-β was downregulated in this group. Furthermore, Usp7 knockdown resulted in a significant increase in the number of TRAP-positive multinuclear cells after stimulation with RANKL for 5 days, compared to those in the NC siRNA group (Fig. 2C and 2D). Meanwhile, the phalloidin staining assay showed a significant increase in the number of F-actin rings in the Usp7 siRNA group stimulated with RANKL for 5 days. Additionally, we seeded BMMs onto the bone slices, and after inducing osteoclast differentiation, these were transfected with Usp7 siRNA. The bone resorption ability of osteoclasts on bone slices was enhanced after treatment Usp7 knockdown, compared to that of the control group (Supplementary Fig. 1). The results suggest that Usp7 silencing in vitro promotes osteoclast formation and bone resorption.

**Figure 3.**
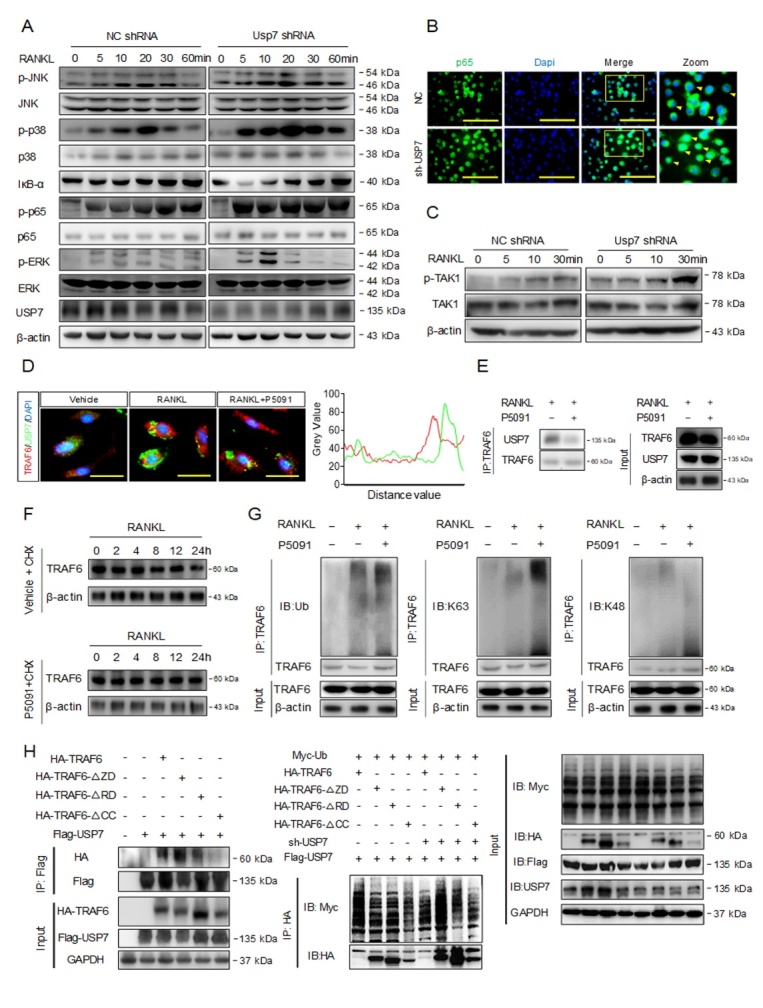
USP7 deubiquitinates TRAF6 in a K63 poly-Ub-dependent manner and merely affects TRAF6-mediated signal transduction. (A) BMMs were transfected with NC shRNA or Usp7 shRNA, and then stimulated with RANKL for the indicated time points. Phosphorylation of JNK, p38, ERK, p65, and degradation of IκBα were analyzed by western blotting. (B) Immunofluorescence staining assay for nuclear translocation of p65 in BMMs. (C) Western blotting was performed to analyze the activation of TAK1 by RANKL in BMMs at the indicated time points. (D) RAW264.7 cells were treated with RANKL or P5091 (a USP7 selective inhibitor), immunofluorescence analysis revealed the co-localization between USP7 and TRAF6. Scale bar =100 um. (E) Co-IP assay was performed to analyze USP7 and TRAF6 binding. (F) Western blotting assay to analyze TRAF6 protein degradation. (G) Co-IP analysis to detect the alterations of K48 or K63 poly-Ub of TRAF6 following RANKL-induced stimulation, with or without P5091. (H) Deletion of the coiled-coil domain (CC), but neither the RING domain (RD) nor the Zinc finger domain (ZD) caused a loss of its ability in TRAF6 to interact with USP7. Deletion of either the TRAF6 RING domain (RD) or the coiled-coil domain (CC) caused an impaired TRAF6 ability to respond to deubiquitination by USP7. All blot assays were repeated three independent experiments.

A previous study employed the USP7-specific inhibitor P5091 to prevent cancer [26], however, the effect of this inhibitor on osteoclasts remains unclear. We first investigated the effect of P5091 on BMM viability (Supplementary Fig. 3A) to determine the appropriate concentration. Subsequently, we treated BMMs with P5091 at different concentrations and observed a dose-dependent promotion of osteoclast formation (Supplementary Fig. 4A). P5091 treatment also accelerated osteoclast formation at different time points (Supplementary Fig. 4C and 4D). Furthermore, following RANKL-induced stimulation, the expression of Nftac1, c-fos, Ctsk, and Atp0d2 was enhanced by P5091 treatment (Supplementary Fig. 5). A luciferase reporter assay performed in BMMs suggested that USP7 inhibition promoted NF-κB and Nfatc1 transcription activities, which are critical for osteoclast differentiation (Supplementary Fig. 6). Taken together, both USP7 knockdown and pharmacological inhibition (P5091) promote osteoclast formation and bone resorption.

We have demonstrated that USP7 knockdown and inhibition promoted osteoclast formation and bone resorption, but it remained unclear whether USP7 expression enhancement had an effect on osteoclastogenesis. Notably, as shown in Figure 2E and 2F, we performed experiments to confirm the efficiency overexpression of Usp7 in the BMMs. Meanwhile, we examined the changes in the OC-related genes and protein expression. Not surprisingly, both OC-related genes and protein expressions decreased upon the Usp7-overexpressing plasmid transfection (Fig. 2E and 2F), but the expression of IFN-β was upregulated in the Usp7-overexpressing plasmid transfection group. Furthermore, by counting for the TRAP-positive cells, we found that Usp7 overexpression using the Usp7-plasmid inhibited osteoclast formation compared with that in the empty vector treatment group. In addition, F-actin staining, and bone resorption assays demonstrated that Usp7 upregulation by the Usp7-plasmid also inhibited osteoclast function (Fig. 2G, 2H and Supplementary Fig. 5). These results were opposite with those of the USP7 expression knockdown, which implied that the balance of USP7 levels is important for osteoclast differentiation and bone resorption for bone homeostasis maintenance. Taken together, USP7 knockdown promotes osteoclast formation and bone resorption, however, USP7 upregulation inhibits osteoclast formation and bone resorption.

### USP7 deubiquitinates TRAF6 in a K63 poly-Ub-dependent manner and merely affects TRAF6-mediated signal transduction

Since USP7 acts as a negative regulator of osteoclast formation and bone resorption, we explored whether USP7 affects the RANKL-dependent NF-κB and MAPKs pathways. BMMs were transfected with empty vectors or shUsp7 and subsequently stimulated with RANKL. Through western blotting analysis, we assessed the protein levels of p38, JNK, ERK, p65, and IκBα phosphorylation. As shown in Figure 3A, these pathways were dramatically upregulated in shUsp7-treated BMMs (Fig. 3A). Nuclear translocation of p65 was also accelerated upon Usp7 silencing (Fig. 3B). Furthermore, these effects were observed in shUsp7-treated BMMs after stimulation with M-CSF (Supplemental Fig. 6). To further explore the upstream pathway, we examined the phosphorylation of transforming growth factor-beta-activated kinase 1 (TAK1), a major member of the MAPK family that exhibits Ser/Thr protein kinase activity. Both NF-κB and MAPK pathways are activated by TAK1-mediated phosphorylation [27]. We observed major TAK1 activation in the shUsp7-treated BMMs (Fig. 3C). These data suggest that USP7 silencing promotes the activation of RANKL-dependent pathways and that the USP7 target molecules could be upstream ubiquitination-related proteins.

A previous study reported that upon RANKL stimulation, TRAF6 recruits Ub proteins from the cytoplasm, triggering the activation of downstream signaling pathways, including the TAK1, NF-κB, and MAPK pathways [28]. This process was also associated with DUBs that could prevent excessive TRAF6 ubiquitination, however, this underlying mechanism remains unclear. Therefore, we explored whether USP7 interacts with TRAF6 to restrict ubiquitination. As shown in Figure 3D and 3E, USP7 and TRAF6 are co-localized in the cytoplasm of BMMs. Further studies showed that USP7 did not affect TRAF6 ubiquitination degradation (Fig. 3F). Subsequently, upon stimulation with RANKL followed by treatment with P5091, we analyzed alterations in Lysine48 (K48 poly-Ub chains) and Lysine63 (K63 poly-Ub chains) of TRAF6. Treatment with P5091 enhanced K63 poly-Ub levels of TRAF6, but not K48 poly-Ub levels (Fig. 3G).

**Figure 4.**
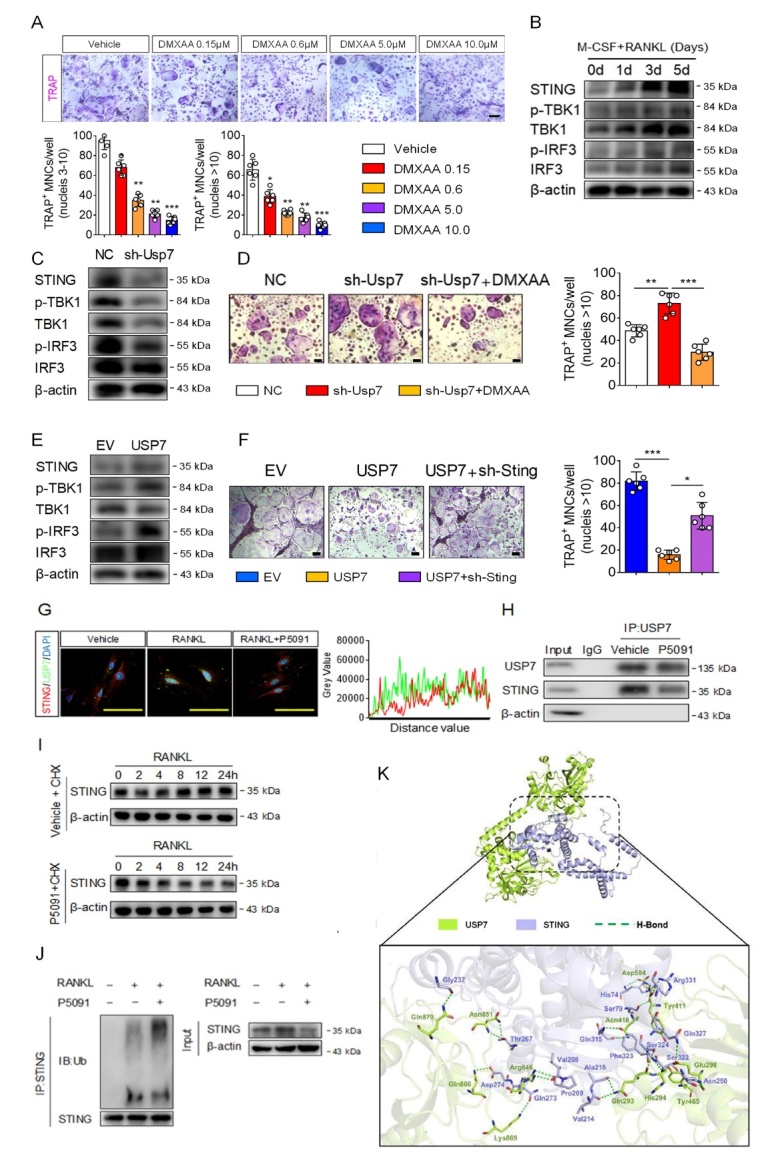
USP7 protects STING against degradation to inhibit osteoclastogenesis. (A) Vadimezan (DMXAA), a STING agonist, inhibited osteoclast formation in a concentration-dependent manner. The cells were fixed and stained for TRAP assay. Scale bar =100 um. The number of TRAP-positive MNCs (3~10 nuclei per well or >10 nuclei per well) per well was analyzed. Data was presented as the mean ± SD. *, P < 0.05. **, P < 0.01. ***, P < 0.005. n=6 per group. Student’s *t*-test analysis was used. (B) Western blotting was performed to analyze the expression of STING, p-TBK1, TBK1, p-IRF3 and IRF3 in osteoclast differentiation under stimulation with DMXA. (C) Western blotting was performed to analyze the activation of STING, TBK1 and IRF3 in the BMMs treatment with Mock or USP7 shRNA. (D) The BMMs were treated with Mock, USP7 shRNA or DMXA for 5 days, then the cells were fixed and stained for TRAP assay. Scale bar =100 um. The number of TRAP-positive MNCs (3~10 nuclei per well or >10 nuclei per well) per well was analyzed. Data was presented as the mean ± SD. *, P < 0.05. **, P < 0.01. n=6 per group. Student’s *t*-test analysis was used. (E) Western blotting was performed to analyze the activation of STING, TBK1 and IRF3 in the BMMs treatment with empty vector or USP7 plasmid. (F) The BMMs were treated with empty vector, USP7 plasmid or Sting shRNA for 5 days, then the cells were fixed and stained for TRAP assay. Scale bar =100 um. The number of TRAP-positive MNCs (3~10 nuclei per well or >10 nuclei per well) per well was analyzed. Data was presented as the mean ± SD. *, P < 0.05. ***, P < 0.005. n=6 per group. Student’s *t*-test analysis was used. (G) The BMMs were treated with M-CSF, RANKL or P5091, immunofluorescence analysis revealed the association between USP7 and STING. Scale bar =100 um. (H) Co-IP assay to detect the alterations between USP7 and STING following RANKL stimulation, with or without P5091. (I) Degradation of STING treatment with P5091 was analyzed Western blot assay under stimulation with RANKL. (J) Alteration of the ubiquitination of STING by treated with P5091. (K) Molecular docking predicting the interaction between USP7 and STING.

TRAF6 has a RING domain at its N-terminus, followed by four zinc finger domains, a coiled-coil (CC) domain, and a TRAF-C domain at the C-terminus [29]. The RING domain is required for interaction with Ubc13 and catalysis of Ub transfer to isopeptide bond formation, which is essential for TRAF6 as an E3 and in signaling [30]. However, instead of longer poly-Ub chains, zinc finger domains play a pivotal role in the synthesis of short poly-Ub chains [13]. In contrast, previous studies have indicated that the CC domain is required for TRAF6 as an E3 and to ensure Ubc13 association with TRAF6 to confer processivity [31]. However, as previously reported, the TRAF-C domain is unlikely to disrupt TRAF6 capability in processive poly-Ub synthesis and signaling [32]. We deleted the three main domains (RING, zinc finger, and CC) essential for TRAF6 poly-Ub chain synthesis and signal transduction. USP7-TRAF6 interaction was disrupted in cells with the deletions of CC, RING, or zinc finger domains (Fig. 4H). Subsequently, we analyzed the effect of USP7 on TRAF6 ubiquitination; the results suggested that USP7 mostly acted on the CC domain in order to deubiquitinate TRAF6 (Fig. 4H).

Altogether, these findings suggest that the TRAF6 RING and CC domains are essential for USP7-mediated deubiquitination, preventing the excessive synthesis of K63 poly-Ub chains and restricting downstream signaling (Fig. 8). Therefore, USP7 regulates osteoclastogenesis to maintain a balance under physiological conditions.

### USP7 protects STING from degradation to inhibit osteoclastogenesis

In previous experiments, we found that alterations in USP7 expression regulated IFN-β mRNA levels. IFN-β, a negative feedback regulator, is produced by the activated stimulator of interferon genes (STING) signaling pathway [33]. Here, we observed a concentration-dependent inhibition in osteoclast formation upon treatment with vadimezan (DMXAA), a STING agonist (Fig. 4A and Supplementary Fig. 3B). Furthermore, during osteoclast differentiation, treatment with DMXAA, further activated the STING/TBK1/IRF3 signaling pathway (Fig. 4B). These observations suggest that excessive activation of the STING signaling pathway inhibits osteoclast differentiation.

Consistent with the previous data, knocking down USP7 promoted osteoclast differentiation, however, it suppressed the STING signaling pathway (Fig. 4C). Furthermore, DMXAA reversed the USP7-induced osteoclast differentiation (Fig. 4D). Conversely, overexpression of USP7 inhibited osteoclast differentiation but activated the STING signaling pathway (Fig. 4E). Simultaneously, knocking down the expression of STING reversed the inhibition of osteoclast differentiation induced by USP7 overexpression (Fig. 4F). However, immunofluorescence and co-IP assays revealed that RANKL induced the association between USP7 and STING in BMMs, while P5091 impaired this effect (Fig. 4G and 4H). Moreover, P5091 impaired USP7-mediated protection of STING degradation (Fig. 4I), occurring an enhanced ubiquitination of STING, for its subsequent degradation (Fig. 4J). While we also performed the molecular docking to predict the interaction between USP7 and STING (Fig. 4K). Collectively, these data suggest that USP7 negatively regulates osteoclast differentiation by preventing STING ubiquitination and degradation.

### Absent or redundant levels of USP7 contribute to the different outcomes of RANKL-induced bone loss in vivo

**Figure 5.**
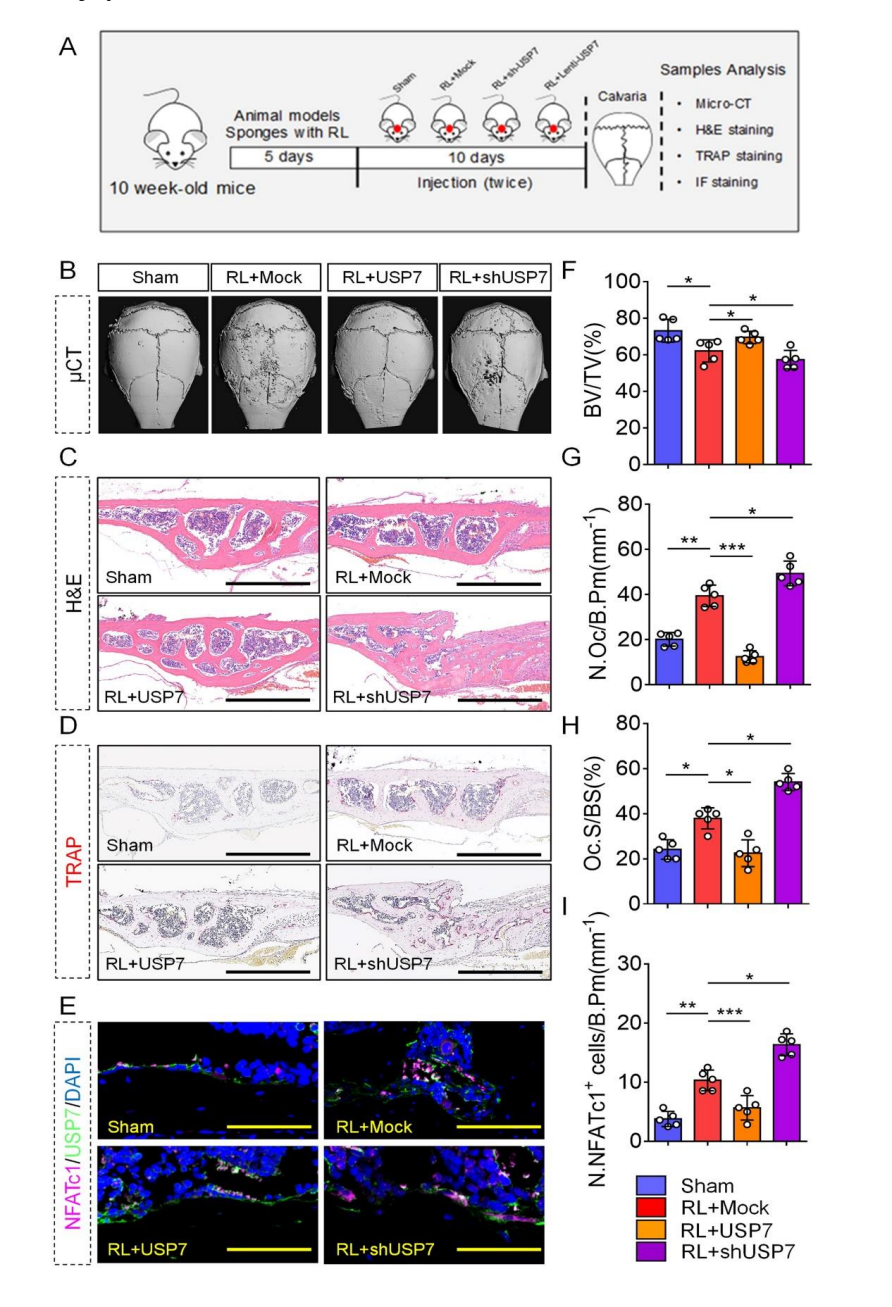
USP7 negatively regulates RANKL-induced bone loss in vivo. (A) Schematic illustration of the establishment of the bone loss mice model and the experimental design to evaluate the effects of USP7. (B) Representative micro-CT image showing calvarial bone resorption in sham-operated mice, and RANKL-soaked sponge with injection of mock, sh-USP7, and AAV-USP7 in mice. (C) Representative H&E staining of the indicated groups. Scale bar = 100 μm. (D) Representative TRAP staining image of the indicated groups. Scale bar = 100 μm. (E) Representative immunofluorescence staining image of NFATc1- and USP7-positive cells in the indicated groups. Scale bar = 100 μm. (F) Bone volume (BV) was reduced in RANKL-treated mice through mock lentiviral injection, compared with sham-operated mice, whereas BV was increased in RANKL-treated mice through AAV-USP7 injection, compared with Mock lentivirus-treated mice. Data are presented as the mean ± SD. * P < 0.05. n=5 per group. one-way ANOVA followed by Tukey’s post hoc analysis was used. (G, H) The number of osteoclast/bone perimeter (N. Oc/B. Pm) and osteoclast surface/bone surface (Oc. S/BS) were analyzed. Data are presented as the mean ± SD. * P < 0.05. **, P < 0.01. ***, P < 0.005. n=5 per group. one-way ANOVA followed by Tukey’s post hoc analysis was used. (I) Quantification of NFATc1 positive cells/bone perimeter. Data are presented as the mean ± SD. * P < 0.05. **, P < 0.01. ***, P < 0.005. n=5 per group. one-way ANOVA followed by Tukey’s post hoc analysis was used.

Next, we addressed whether USP7 showed a similar effect on osteoclastogenesis in vivo. Reproducing our previous study [24], we treated mice with RANKL-enriched sponges on the skull surfaces for 10 days (Fig. 5A). Meanwhile, we injected lenti-USP7 and sh-USP7 with sponges onto the bone surface or saline as a control. After the mice were sacrificed, we analyzed the calvarial bones by micro-CT, H&E, TRAP staining, and immunofluorescence. Micro-CT data demonstrated that the lenti-USP7-treated calvarial bone group had an increase in BV/TV compared with that in the mock-treated group, however, the bone resorption areas of the lenti-USP7 group were reduced (Fig. 5B, 5C, and 5F). In contrast, the sh-USP7-treated calvarial bone group showed a decrease in BV/TV compared to the control group. Furthermore, TRAP staining revealed a lower number of osteoclasts and a lower osteoclast surface percentage in the calvarial bone of the lentivirus-USP7 treated group, while opposite results were observed in the sh-USP7 group (Fig. 5D, 5G, and 5H). Consistent with previous data, RANKL treatment induced the expression of USP7 and NFATc1. However, USP7 overexpression attenuated the levels of NFATc1 (Fig. 5E and 5I). Taken together, USP7 plays a determinant role in bone maintenance in vivo, its overexpression attenuates bone loss and its knockdown accelerates this process.

### OVX mice show reduced USP7 in the bone

**Figure 6.**
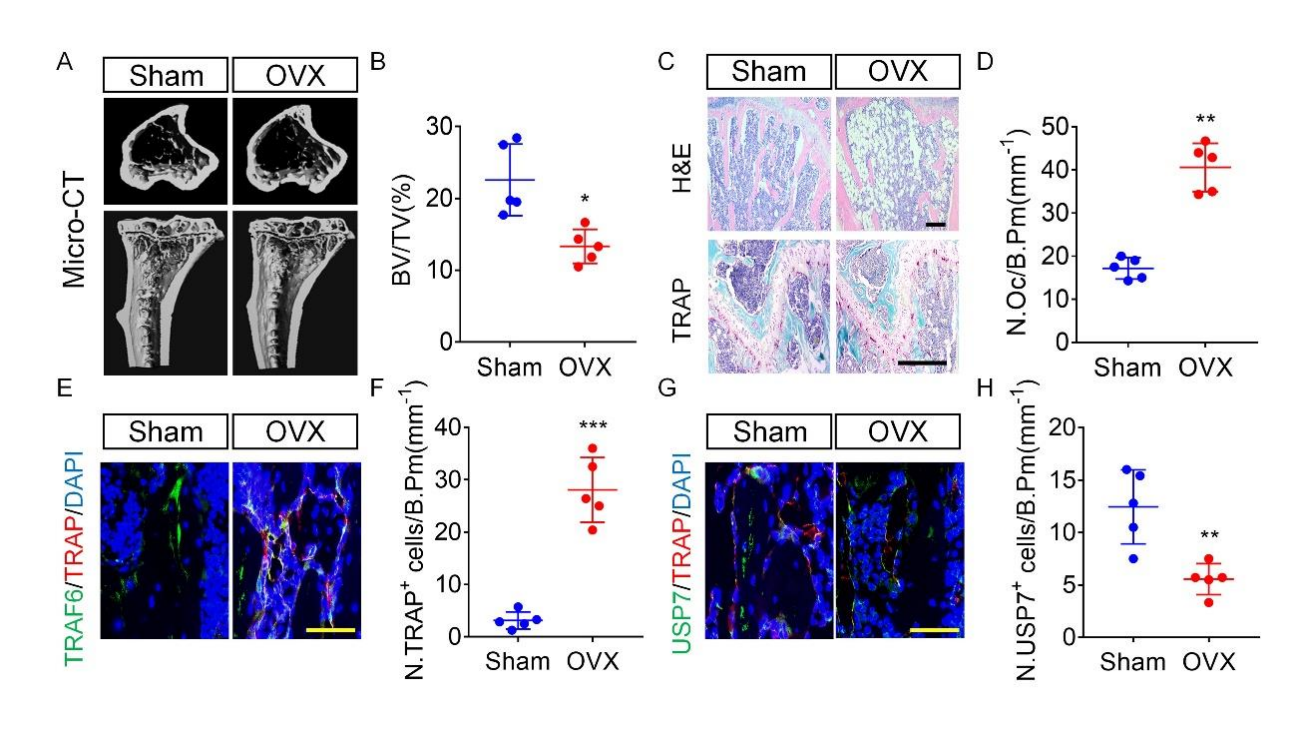
USP7 expression is decreased in the bone sections of osteoporotic mice. (A) Representative micro-CT image showing trabecular bone loss in OVX mice compared with sham-operated mice. (B) Bone total volume (BV/TV) was reduced in OVX mice. Data are presented as the mean ± SD. * P < 0.05. n=5 per group. Student’s *t*-test analysis was used. (C) Representative H&E staining and TRAP staining of trabecular bone in OVX mice compared with sham-operated mice. Scale bar = 100 μm. (D) The number of osteoclast/bone perimeter (N. Oc/B. Pm) was analyzed. Data are presented as the mean ± SD. **, P < 0.01. n=5 per group. Student’s *t*-test analysis was used. (E, F) Representative immunofluorescence staining images of TRAP-, USP7-, and TRAF6-positive cells in OVX mice compared with sham-operated mice. Scale bar = 100 μm. n=5 per group. Student’s *t*-test analysis was used. (G, H) Quantification of TRAP- and USP7-positive cells/bone perimeter. Data are presented as the mean ± SD. * P < 0.05. **, P < 0.01. n=5 per group. Student’s *t*-test analysis was used.

Osteoporosis is one of the most common bone metabolism disorders, characterized by excessive bone resorption or weakened bone formation [7]. To evaluate the potential role of USP7 in bone metabolism in vivo, we performed ovariectomy in mice, as an animal model of postmenopausal osteoporosis. The micro-CT and H&E staining revealed a significant reduction of trabecular bone in OVX mice, while the TRAP staining showed an increased number of osteoclasts in these mice, compared to the control group (Fig. 6A-6D). These results support the OVX-induced bone loss. Subsequently, we examined the expression of TRAP, TRAF6, and USP7 in bone sections using immunofluorescence (Fig. 6E and 6F). USP7 expression was decreased in the TRAP^+^ osteoclast lineage of OVX mice, compared to sham-operated mice (Fig. 6G and 6H). In summary, these results indicate that in osteoporosis USP7 expression is altered in osteoclast lineage cells, which might contribute to the disease phenotype.

**Figure 7.**
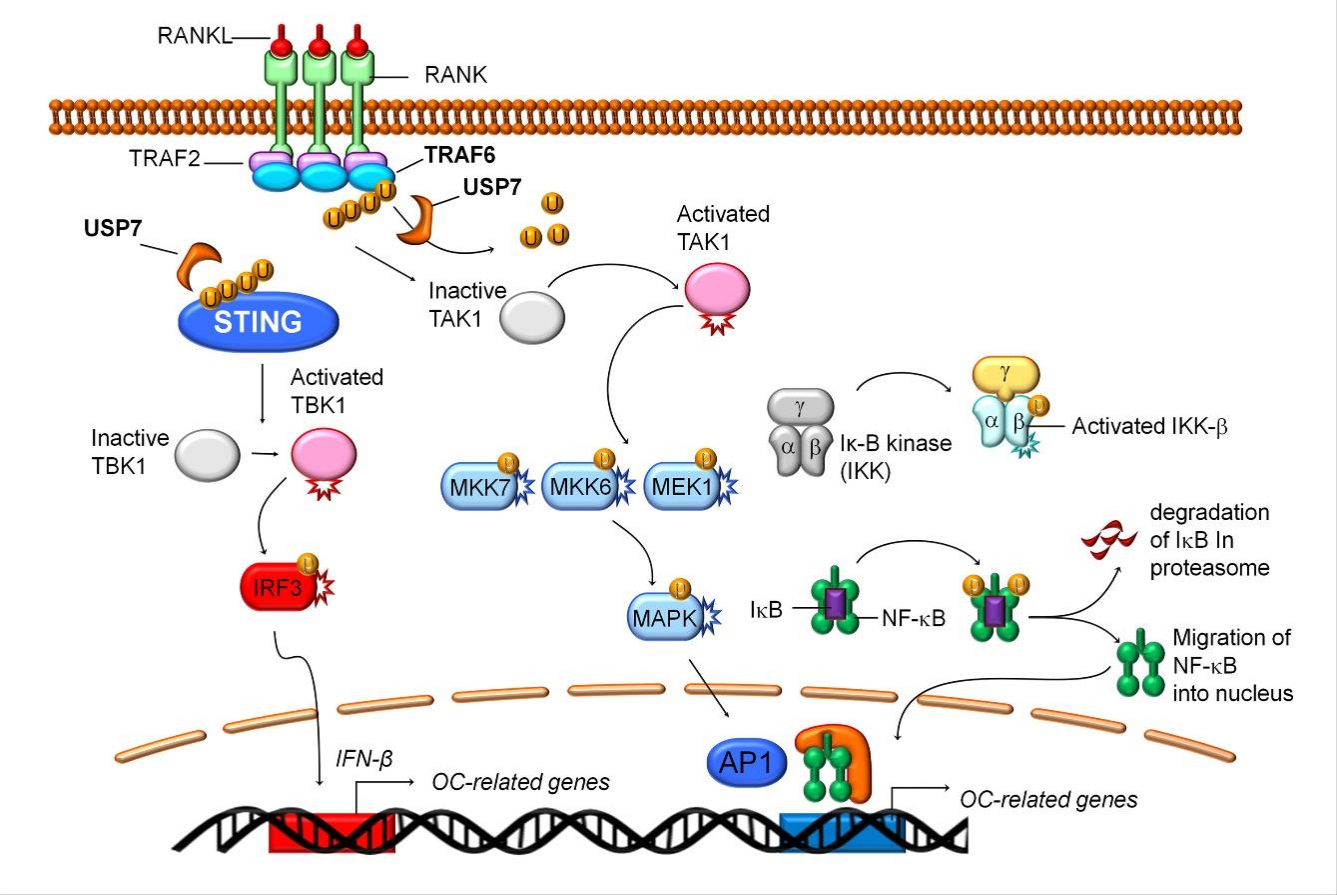
Schematic representation of the functions of USP7 on osteoclast differentiation. USP7 negatively regulated the osteoclastogenesis process in BMMs by acting as a deubiquitination enzyme to mediate the K63-linked ubiquitination of TRAF6 at the RING domain and CC domain, and mediate the degradation of STING to promote the expression of IFN-β.

## DISCUSSION

Negative regulation of excessive osteoclast formation and bone resorption is determinant for maintaining bone homeostasis and preventing the development of osteoclast-related diseases. Both the initiation and termination of signaling pathways underlying the formation of mature osteoclasts are critical checkpoints that limit excessive bone resorption [24, 35]. The complex network of RANKL-response factors, including Irf8, Stac2, Pax6, and extracellular secreted molecules, such as interferon-β (IFN-β), controls bone homeostasis [24, 35, 36]. In this study, we have screened a large number of ubiquitin-specific peptidases (USPs) to explore a novel target for preventing excessive osteoclast formation (data not shown). DUB separates Ub from the targeted protein and Ubs in the poly-Ub chains [37] and have been implicated in cell differentiation, cell metabolism, and organ homeostasis [38]. We found ubiquitin-specific protease 7 (USP7) to be significantly increased by the stimulation with M-CSF and RANKL in BMMs. Moreover, knockdown or inhibition of USP7 accelerated osteoclast formation and bone resorption. In contrast, USP7 overexpression repressed osteoclast formation and bone resorption. Consistent with the in vitro results, lenti-USP7 administration attenuated RANKL-induced bone loss in calvarial bones in vivo. These results suggest that USP7 negatively regulates osteoclast formation and bone resorption.

The regulation of RANKL-activated signaling pathways can be divided into three phases, including initiation, commitment, and termination, owing to the signaling process of osteoclast differentiation [39]. In the initiation phase, treatment with RANKL results in the rapid activation of TAK-1, MAPKs, and canonical NF-κB signaling pathways via ubiquitination of TRAF6 [28]. Subsequently, in the commitment phase, NFATc1 accumulates and initiates Ca^2+^ oscillations [40]. Finally, in the termination phase, RANKL-induced signaling regulates cell fusion and bone resorption via NFATc1-mediated transcription of osteoclast-specific genes [41]. Considering that USP7 is increased during osteoclast differentiation, we hypothesized that USP7 mainly affects the initiation phase to the termination phase of the osteoclast differentiation signaling process. Further analysis of the underlying mechanism revealed that knocking down USP7 promotes the activation of TAK-1, MAPKs, and canonical NF-κB signaling pathways in response to RANKL stimulation. RANKL stimulates K63 poly-Ub ubiquitination of TRAF6 [42]. Therefore, we speculated that excessive K63 ubiquitination of TRAF6 contributes to the activation of these downstream signaling pathways upon USP7 knockdown. In HSV-activated THP1 cells USP7 promotes de-ubiquitination of TRAF6 [43]. Moreover, Xiang et al. reported that USP7 regulates K63-linked polyubiquitination of TRAF3 and TRAF6 [44]. Consistent with these reports, our data suggest that USP7 regulates the deubiquitination of TRAF6 by targeting K63 poly-Ub chains, restricting signal transduction during osteoclast differentiation. Ubiquitination of TRAF6 mainly occurs in the RING, zinc finger, and CC domains [45]. Stimulation with RANKL recruits TRAF6 and poly-Ub chains to initiate a signaling cascade related to these key domains [46]. Our results revealed that the de-ubiquitination effect of USP7 mainly depends on the RING and CC domains of TRAF6. Through the regulation of the de-ubiquitination of TRAF6, USP7 acts as a negative regulator of osteoclast formation and bone resorption. However, the mechanism through which USP7 or other DUBs regulate the commitment and termination phases of osteoclast differentiation requires further investigation.

In this study, we also uncovered that the knockdown of Usp7 suppressed the mRNA expression of IFN-β, whereas overexpression of Usp7 promoted its increase. A previous study suggested that IFN-β in osteoclasts is involved in the negative feedback regulation pathway [20]. Furthermore, the evidence suggests that IFN-β is produced by the activated STING signaling pathway [21]. IFN-β can bind to the IFN-α/β receptor to activate various factors that regulate downstream signaling pathways [22]. During osteoclast differentiation, mitochondrial biogenesis is upregulated, therefore increasing mitochondrial DNA (mtDNA) [47, 48]. The mtDNA can promote the intrinsic activation of STING, therefore increasing IFN-β expression. Using the STING agonist, DMXAA, we inhibited osteoclast differentiation in a concentration-dependent manner. Simultaneously, in RANKL-stimulated cells treated with DMXAA, the STING/TBK1/IRF3 signaling pathway was activated. These data suggest that activation of STING signaling might be an intrinsic negative regulatory pathway of osteoclast differentiation; however, excessive activation of STING signaling in BMMs suppresses osteoclast formation. Furthermore, by knocking down or overexpressing USP7, we found STING signaling pathway suppressed or activated, respectively. The immunofluorescence and co-IP evaluations also provided evidence of the interaction between USP7 and STING, and how it regulates the negative signaling pathway to induce IFN-β expression. Combined with the TRAF6 signaling pathway, USP7 negatively regulates osteoclast differentiation by hybrid mechanisms, which are mediated by K63 ubiquitination of TRAF6-induced signaling transduction and STING-induced IFN-β expression.

Further exploring the pathophysiological role of USP7 in bone, we uncovered that USP7 is abnormally decreased in osteopenia mice. Previous studies have demonstrated that abnormal USP7 expression plays an important role in the pathogenesis of inflammatory responses, and nasopharyngeal, lung, and cervical cancers [49-51]. USP7 has been shown to positively regulate osteogenesis, both in vitro and in vivo [52]. Based on our results and those of other studies, USP7 might negatively regulate osteoclast differentiation but positively regulate osteogenesis. The abnormal decrease in USP7 expression found in osteoporosis further supports its critical role in the pathogenesis of bone-related diseases. Further studies focusing on the upregulation of USP7 may provide novel insights for clinical applications.

Since the approval of the first-in-class proteasome inhibitor bortezomib (Velcade®) by the Food and Drug Administration for treating relapsed multiple myeloma in 2003 [53], an increasing number of research institutions and pharmaceutical companies have developed simplified and efficient chemical synthesis protocols to identify compounds targeting USP7 with affinity, specificity, and stability [54, 55]. However, despite extensive investigation of USP7 inhibitors in the field of tumor therapy, little is known about their application in the treatment of bone-related diseases. In this study, we observed that P5091, a selective USP7 inhibitor, promotes osteoclast formation, bone resorption, and osteoclast-specific gene expression. Therefore, USP7 inhibitors may serve as potential therapeutic agents for heterotopic ossification. Considering that P5091 has been regarded as a potential anticancer drug and that our results suggest that this compound may accelerate osteoclast formation and bone resorption; the use of this drug might account for side effects such as osteoporosis during anticancer therapy. Tang et al. reported a similar case, where HBX41108 and P5091 inhibited osteogenesis, which also provided a potential therapeutic approach for hyperplasia of bone formation, however, resulted in the side effect of osteoporosis [52]. There is an increasing interest in the development of USP7-enhancing compounds for the treatment of bone-related diseases, nevertheless, the side effects of these drugs should be considered.

In conclusion, by identifying USP7 as a negative regulator of RANKL-RANK signaling in osteoclasts, the present study provides novel insights into the hybrid mechanism of osteoclast differentiation. Upon RANKL stimulation, USP7 de-ubiquitinates TRAF6 at the RING and CC domains, resulting in the suppression of the TAK-1, MAPKs, and canonical NF-κB signaling pathways. USP7 also promotes the stability of STING to induce IFN-β expression. Moreover, P5091, a USP7 inhibitor, accelerates osteoclast formation and bone resorption, suggesting that the side effects of osteoporosis should be considered while using this compound in anticancer therapy. Additionally, the decreased expression of USP7 in osteopenia mice suggests a critical role in abnormal bone homeostasis and disease. These results demonstrate that USP7 negatively regulates osteoclast differentiation via a hybrid mechanism, in which de-ubiquitination-mediated TRAF6 signal transduction and de-ubiquitination mediate the degradation of STING. We propose that USP7 could be used as a potential biocompatible strategy for the treatment of bone diseases caused by excessive osteoclast activity.

## Supplementary Materials

The Supplementary data can be found online at: www.aginganddisease.org/EN/10.14336/AD.2023.0325-1.
