# Supplementary material for: USP7 Inhibits Osteoclastogenesis via Dual Effects of Attenuating TRAF6/TAK1 Axis and Stimulating STING Signaling
Source: Aging Dis. 2023 Dec 1;14(6):2267–83. doi: 10.14336/AD.2023.0325-1 (PMC10676781; doi:10.14336/AD.2023.0325-1)
Supplement: Supplementary file 1 — The Supplementary data can be found online at: www.aginganddisease.org/EN/10.14336/AD.2023.0325-1. [file AD-14-6-2267-s.pdf]

USP7 Inhibits Osteoclastogenesis *via* Dual Effects of Attenuating TRAF6/TAK1 Axis and Stimulating STING Signaling

Ziang Xie[1,2#,*], Yizheng Wu[1,2#], Yang Shen[1,2#], Jiandong Guo[3], Putao Yuan[1,2], Qingliang Ma[1,2], Shiyu Wang[1,2], Zhiwei Jie[1,2], Hongyi Zhou[1,2], Shunwu Fan[1,2,*], Shuai Chen[1,2*]

© 2023. Yang J et al. Published online athttp://www.aginganddisease.org/EN/10.14336/AD.2023.0325-1

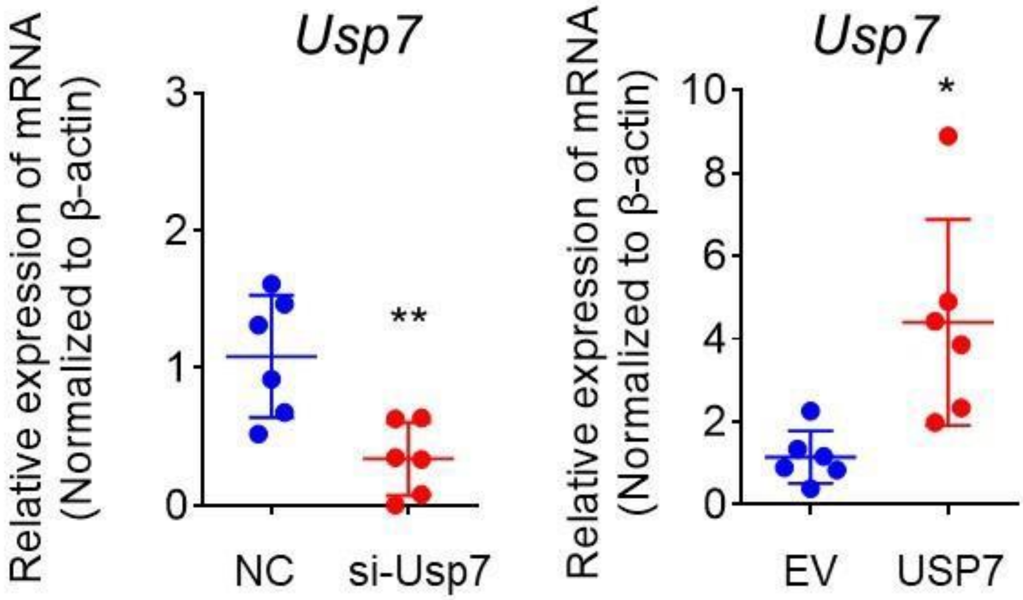

Supplemental Figure 1. The efficiency of silencing USP7 and overexpression of USP7. The effect of Usp7 sirRNA or overexpression plasmid of USP7 on BMMs was analyzed by qPCR assay. Data are presented as the mean ± S.D. *, P<0.05. **, P<0.01.

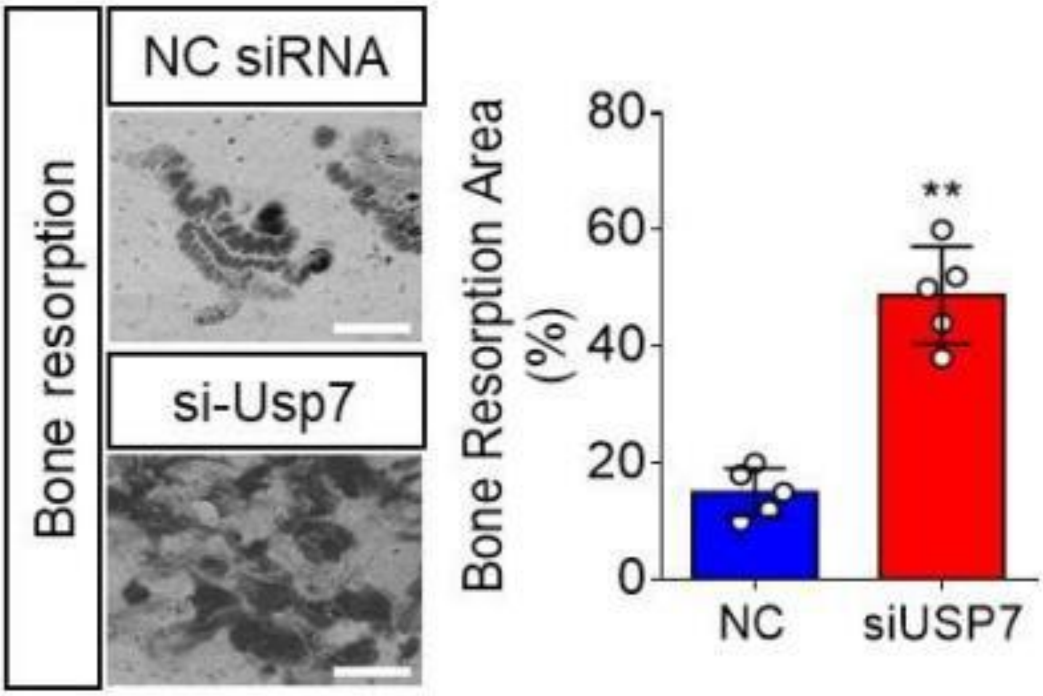

Supplemental Figure 2. The effects of Usp7 siRNA on bone resorption. The effects of NC siRNA or Usp7 siRNA on bone resorption was analyzed by bone slices assay. Scale bar=100 μm. Data are presented as the mean ± S.D. **, P<0.01.

© 2023. Yang J et al. Published online at http://www.aginganddisease.org/EN/10.14336/AD.2023.0325-1

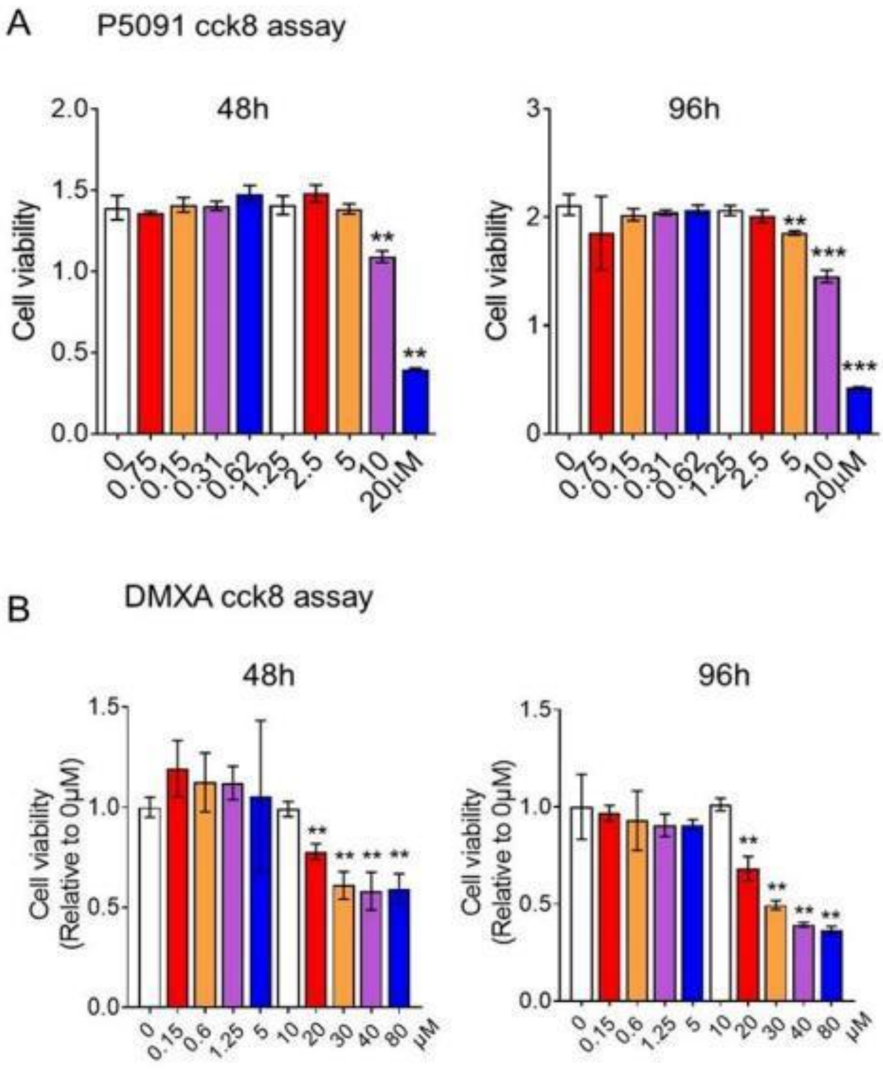

Supplemental Figure 3. Cell viability of P5091 on BMMs. (A) The effect of P5091 on BMMs was analyzed by CCK8 assay for treatment with 48h or 96h. Data are presented as the mean ± S.D. *, P<0.05. **, P<0.01.

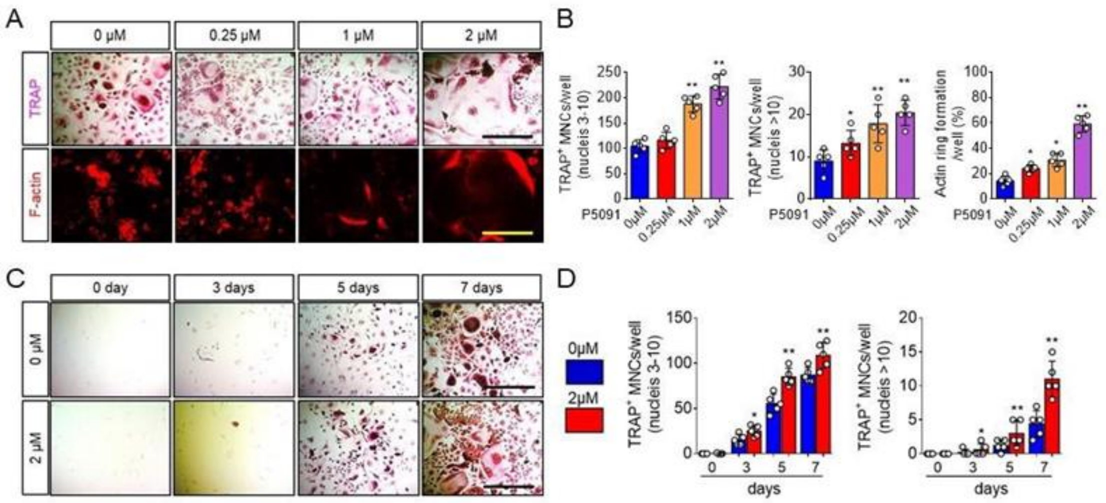

Supplemental Figure 4. The effects of P5091 on osteoclast formation in dose- dependent manner. (A) The effect of different concentrations of P5091 on BMMs was analyzed by TRAP and F-actin assay for treatment 5 days. (B) Quantitative analeysis of osteoclast number. (C) The effect of different days of P5091 on BMMs was analyzed by TRAP and F-actin assay for treatment with 2μM. (D) Quantitative analeysis of osteoclast number. Data are presented as the mean ± S.D. *, P<0.05. **, P<0.01.

© 2023. Yang J et al. Published online at http://www.aginganddisease.org/EN/10.14336/AD.2023.0325-1

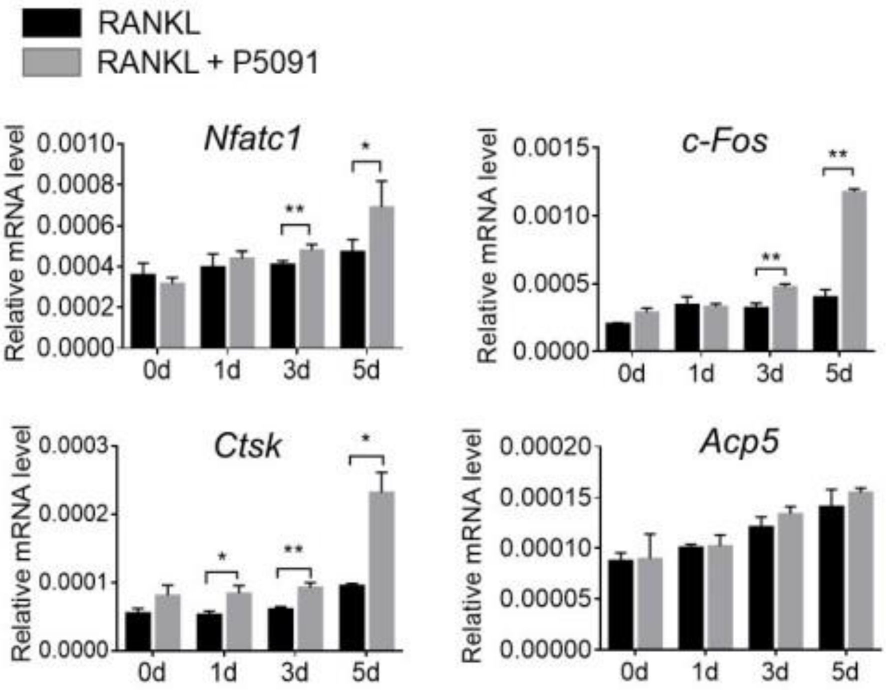

Supplemental Figure 5. The effects of P5091 on osteoclast related genes expression. In stimulation with RANKL, the expression of Nfatc1, c-fos, Ctsk and Atp0d2 in RAW264.7 cells were analyzed by qPCR assay in treatment with P5091 (2 μM) or not. Data are presented as the mean ± S.D. *, P<0.05. **, P<0.01.

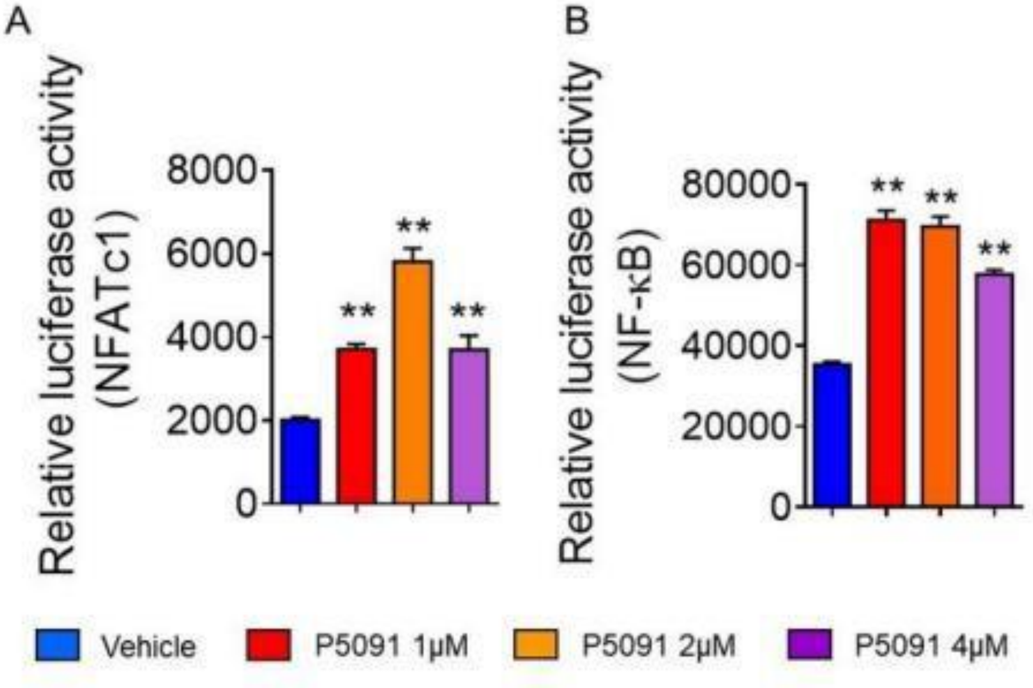

Supplemental Figure 6. The effects of P5091 on transcription activity of Nfatc1 and NF-κB. (A) In the presence of M-CSF and RANKL, the BMMs were treated with different concentrations of P5091, the transcription activity of Nfatc1 was tested by dual luciferase reporter genes system. Data are presented as the mean ± S.D. **, P<0.01. (B) In the presence of M-CSF and RANKL, the BMMs were treated with different concentrations of P5091, the transcription activity of NF-κB was tested by dual luciferase reporter genes system. Data are presented as the mean ± S.D. **, P<0.01.

© 2023. Yang J et al. Published online at http://www.aginganddisease.org/EN/10.14336/AD.2023.0325-1

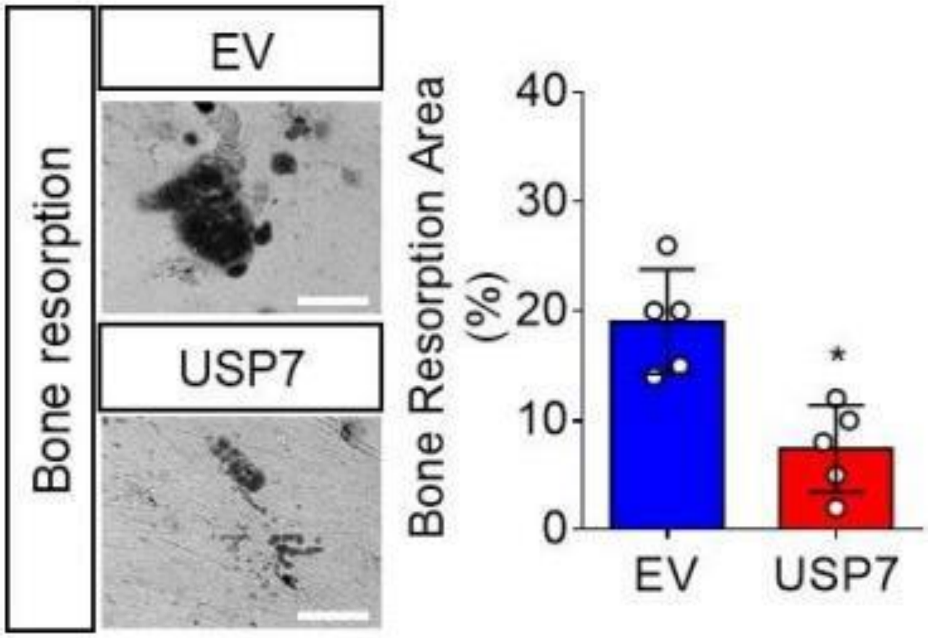

Supplemental Figure 7. The effects of Usp7 overexpress-plasmid on bone resorption. The effects of empty vector or Usp7 plasmid on bone resorption was analyzed by bone slices assay. Scale bar=100 μm. Data are presented as the mean ± S.D. *, P<0.05.

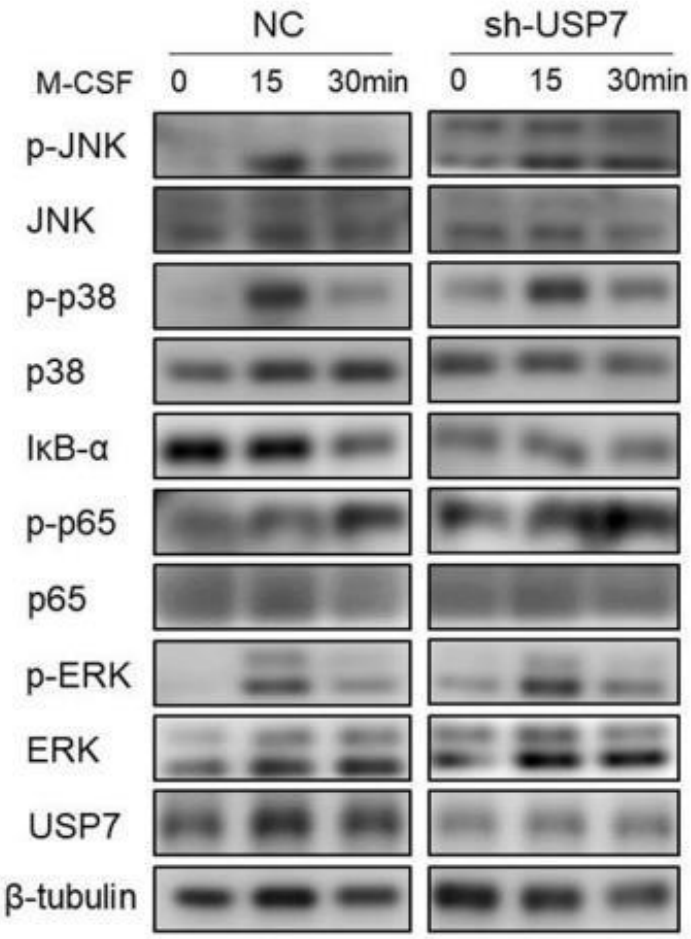

Supplemental Figure 8. The effects of sh-USP7 on MAPKs and NF-κB signaling pathways in stimulation with M-CSF. In stimulation with M-CSF at different time points,

© 2023. Yang J et al. Published online athttp://www.aginganddisease.org/EN/10.14336/AD.2023.0325-1

the activation alteration of MAPKs and NF-κB signaling pathways by treatment with sh-USP7

© 2023. Yang J et al. Published online at http://www.aginganddisease.org/EN/10.14336/AD.2023.0325-1

Supplementary Table 1. The detailed information for antibodies.

Regent	Source	Dilution
Anti-GAPDH	Abcam	WB 1:2500
Anti-β-actin	Abcam	WB 1:2500
Anti-USP7	Cell Signaling Technology, ProteinTech	WB 1:1000
Anti-NFATc1	Cell Signaling Technology	WB 1:1000
Anti-c-Fos	Cell Signaling Technology	WB 1:1000
Anti-CTSK	Santacruz, Abcam	WB 1:500, IF 1:200
Anti-TRAP	GeneTex, Abcam	WB 1:1000
Anti-TRAF6	Santacruz, Abcam	WB 1:1000, IF 1:200
Anti-Ub	Cell Signaling Technology	WB 1:1000
Anti-Ub(K63)	Cell Signaling Technology	WB 1:1000
Anti-Ub(K48)	Cell Signaling Technology	WB 1:1000
Anti-HA	Abcam	WB 1:5000
Anti-Flag	Abcam	WB 1:5000
Anti-Myc	Abcam	WB 1:5000
Anti-IκBα	Cell Signaling Technology	WB 1:1000
Anti-Phospho- TAK1	Cell Signaling Technology	WB 1:1000
Anti-TAK1	Cell Signaling Technology	WB 1:1000
Anti-Phosphor-p38	Cell Signaling Technology	WB 1:1000
Anti-Phospho-JNK	Cell Signaling Technology	WB 1:1000
Anti-Phospho-ERK	Cell Signaling Technology	WB 1:1000
Anti-Total-p38	Cell Signaling Technology	WB 1:1000
Anti-Total-JNK	Cell Signaling Technology	WB 1:1000
Anti-Total-ERK	Cell Signaling Technology	WB 1:1000

© 2023. Yang J et al. Published online athttp://www.aginganddisease.org/EN/10.14336/AD.2023.0325-1

SUPPLEMENTARY DATA

Supplementary table 2. Primers for quantitative RT-PCR.

Genes	Upstream (5′-3′)	Downstream (5′-3′)
Gapdh	ACCCAGAAGACTGTGGATGG	CACATTGGGGGTAGGAACAC
Ctsk	CTTCCAATACGTGCAGCAGA	TCTTCAGGGCTTTCTCGTTC
Acp5	CTGGAGTGCACGATGCCAGCGAC A	TCCGTGCTCGGCGATGGACCAG A
c-Fos	CCAGTCAAGAGCATCAGCAA	AAGTAGTGCAGCCCGGAGTA
Nfatc1	CCGTTGCTTCCAAAAATAACA	TGTGGGATGTGAACTCGGAA
Usp7	GGTGGAATTCCTTGGAGTCTGT	AGTCCTCATTGTCTTGCCTGTT

Supplementary Table 3. siRNA sequences used in our study.

Alias	sense (5′-3′)	antisense (5′-3′)
si-Usp7#1	GAGGCAACCUUUCAGUUCAT T	UGAACUGAAAGGUUGCCUCTT
si-Usp7#2	GCGUCCCUUUAGCAUUACAT T	UGUAAUGCUAAAGGGACGCTT
si-Usp7#3	GCCCAAGUUUGAUAAAGAUT T	AUCUUUAUCAAACUUGGGCTT

© 2023. Xie Z et al. Published online at http://www.aginganddisease.org/EN/10.14336/AD.2023.0325
